# Quantum-enhanced generative artificial intelligence: a critical review of classical limitations, complexity barriers, and hybrid quantum–classical architectures

**DOI:** 10.3389/frai.2026.1836641

**Published:** 2026-07-21

**Authors:** Diljot Singh, Omana J., Smrithy G. S.

**Affiliations:** School of Computer Science and Engineering, Vellore Institute of Technology, Chennai, India

**Keywords:** generative AI, Grover's search algorithm, hybrid quantum-classical architecture, large language model, quantum probabilistic model, retrieval-augmented generation, variational quantum eigensolver

## Abstract

The rapid commercialization of generative artificial intelligence (AI), along with the maturation of quantum technologies has raised a question: can quantum-powered neural networks become the next major shift in large language model (LLM) technology? This naturally leads to another misconception that quantum systems will replace classical LLMs. In this study, both architectures are compared in a contrastive manner in terms of mathematics. The data reveals that identical dynamics that help classical systems learn natural language distributions constrain its ability to use efficient sampling of quantum-mechanical spaces. Performing complexity-theoretic separations (i.e., the widely believed but unproven conjecture that BPP ⊆ BQP) and a 2025 preprint reporting experimental demonstrations of quantum advantage for generative tasks we conclude that quantum utility is unlikely to lie in tasks involving natural language processing under current architectures, but rather in certain computational subroutines. We then suggest a hybrid quantum-classical architecture as the best direction to take in the future, as it has the advantages of both paradigms. This is done by studying a case study that optimizes retrieval-augmented generation (RAG) pipelines with Grover's search algorithm.

## 1 Introduction

The quantum language models (QLMs) employ quantum circuits to process and generate sequences, leveraging the principles of superposition and entanglement in quantum computing; however, large language models (LLMs) are classical neural networks that are trained on a large body of text using backpropagation (Rumelhart et al., 1986) and gradient descent (Ruder, 2016). As the market rapidly evolves around generative AI, with industry studies predicting transformative economic impact (McKinsey & Company., 2025), and quantum technology rapidly matures, the possibility and potential of merging the two paradigms has captured attention. More generally, machine learning and quantum computing are joining forces in quantum-enhanced optimization, classification and generative modeling (Biamonte et al., 2017; Dunjko and Briegel, 2018). The classical transformer paradigm has taken the forefront of NLP with modern LLMs like GPT-3 (Brown et al., 2020) showing outstanding few-shot learning abilities.

It is important to acknowledge that there are a number of misconceptions and buzzwords associated with the Quantum topics, which need to be resolved before proceeding. Many people think that QLMs will replace classical LLMs in generative AI applications. This is a wrong assumption. Currently, QLMs do not outperform LLMs nor are they likely to do so for natural language tasks. Classical LLMs are designed to process natural data: Text, Images, Audio and Video data. Transformer models have key design elements that capture the statistical characteristics of data from the NL and sensory modalities, including self-attention (Vaswani et al., 2017) and positional encoding. QLMs are not arithmetically designed to perform these tasks. The problems with quantum mechanical structure, on the other hand (Biamonte et al., 2017; Schuld et al., 2015; Schuld and Petruccione, 2021) are most naturally handled by quantum machine learning, and have been much confused in the popular literature, to my knowledge.

**Figure 1:**
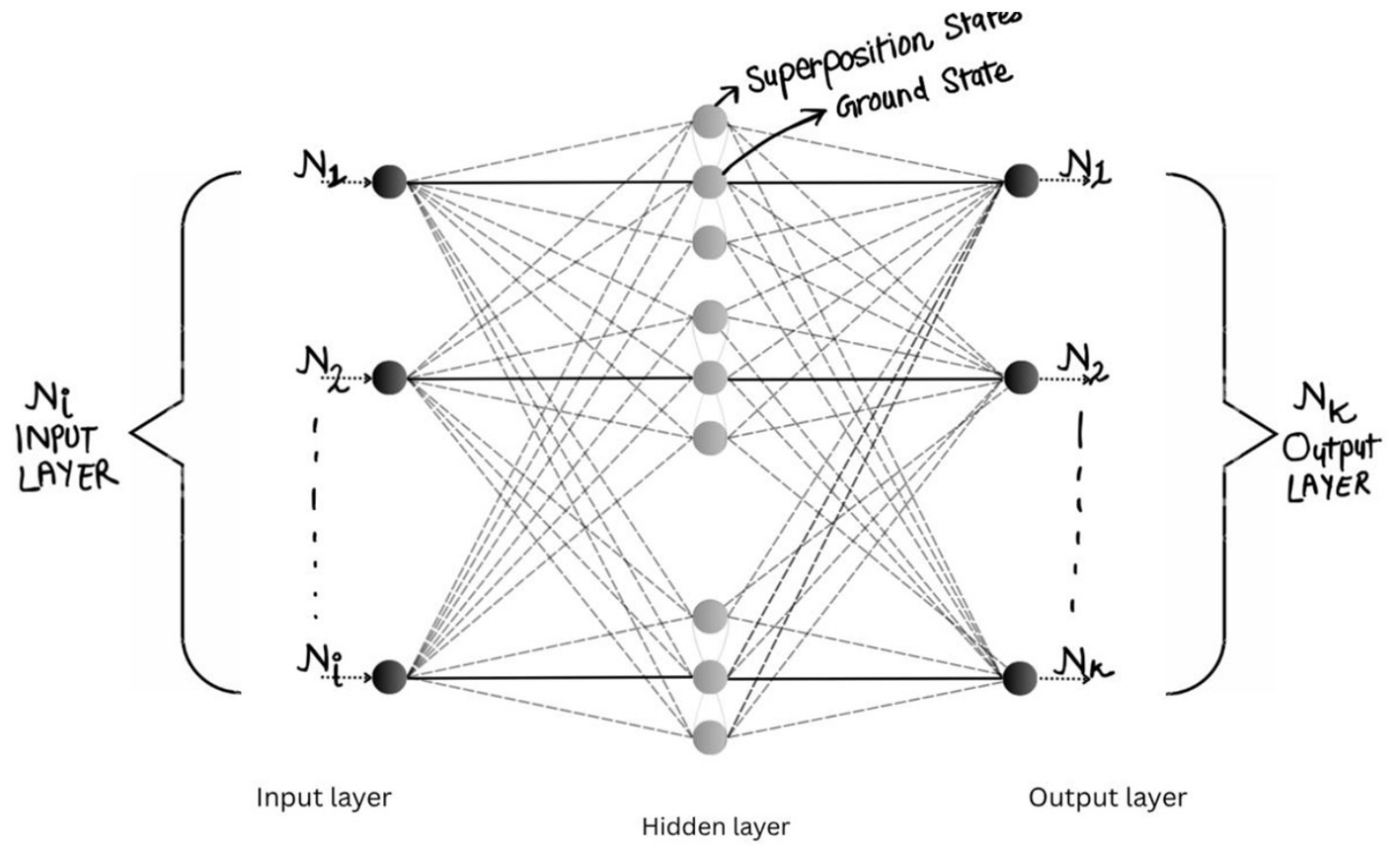
The QLM architecture.

To wrap up with that original question: If the quantum system doesn't enhance natural language generation, what makes them a consideration for generative AI applications in general? The solution is to understand the limitations of mathematical problems in classical computation. Two typical classes of problems that are difficult for classical computational methods are quantum-mechanical probability distributions like molecular wavefunctions and exponentially large combinatorial optimization spaces (Preskill, 2018) such as constraint satisfaction and high dimensional search problems. Figure 1 shows the structural difference between a QLM using superposition states and a classical neural network. Classical models work well with natural data distributions, but are not effective at efficiently sampling from the output of a quantum circuit or for exploring the space of combinations.

It's a distinction that's essential: quantum computing won't help with tasks like creating images and text. Rather, it is intended to solve problems which are fundamental efficiency problems for classical systems within known complexity theory.

## 2 Review methodology

This review has been conducted via a search of existing literature using Google Scholar, IEEE Xplore, ACM Digital Library, and arXiv. Search terms included combinations of: “quantum generative models,” “quantum machine learning,” “large language models,” “hybrid quantum-classical architectures,” “variational quantum eigensolver,” “Grover's algorithm,” “retrieval-augmented generation,” “barren plateaus,” “quantum supremacy,” and “NISQ.” Papers are included only and only if they (a) were peer-reviewed publications or widely cited preprints, (b) were published between 1994 and 2026, and (c) were directly relevant to at least one of the three main themes of this review (classical generative AI limitations, quantum computational advantage, hybrid quantum-classical architectures). Gray literature: (including press releases and industry whitepapers) is used only where no peer-reviewed equivalent exists and is explicitly flagged as such in the text.

## 3 Theoretical foundations and limitations of classical generative methods

Classical models face fundamental scaling barriers when a quantum system generates a probability distribution or when the state space expands exponentially. This limitation arises primarily from the mathematical design of classical models rather than hardware constraints alone.

### 3.1 Quantum sampling complexity

Suppose that the d gates are placed in a quantum circuit *U* acting on n qubits, in the state |0^n^, the state in the computational basis. According to the Born rule (Nielsen and Chuang, 2010) the probability of observing outcome x is given by Equation 1:


$$\begin{array}{l} p\left(x\right)\;=\;|\langle x|U|0^{\text{n}}\rangle {|}^{2} \end{array}$$ (1)

where x ε {0,1}^n^ is a computational basis state and 〈 x|*U*|0^n^〉 is the probability amplitude. “Born supremacy” (Coyle et al., 2020) refers to the hardness of performing classical sampling from Born rule distributions, and is the theoretical basis of quantum generative modeling and the motivation for the quantum advantage in some generative tasks.

The main computational problem is to follow quantum interference effects. A single amplitude 〈x|U|0^n^〉 is computed by keeping the quantum state vector, which has 2n complex probability amplitudes. For a small 50-qubit system, this amounts to 250 or so complex numbers per qubit, or 16 bytes per complex number, for a total of 250 × 16 bytes per qubit = 18 petabytes of memory. This is much more than the memory of any current supercomputer—a limit not intrinsic to engineering but to the exponential size of the Hilbert space, as mentioned in regard to the “entanglement frontier” (Preskill, 2012).

This limitation is not a consequence of the mediocre algorithm, but an exponential increase in the size of quantum state space. Even on the classical machine, the complexity of sampling from a quantum circuit distribution is O(2^n^·poly(d)), which makes it hopeless to sample more than about 40–50 qubits even with future enhancements to classical machines.

By physically running the circuit, however, a physical quantum computer produces independent samples from this distribution in time *O(d)*. There are no explicit 2^n^ amplitudes in the quantum system that are computed or stored. The quantum state itself is instead physically realized by superposition, and measurement collapses the quantum state to give samples in a probabilistic manner according to the Born rule.

### 3.2 Complexity-theoretic separations

Under the widely held but unproven conjecture that BPP ⊆ BQP—that bounded-error probabilistic polynomial time is strictly contained within bounded-error quantum polynomial time—there exist probability distributions that quantum computers can efficiently sample from while classical computers probably cannot generate samples from in polynomial time. It is essential to emphasize that BPP ⊆ BQP remains a conjecture, not an established theorem; all arguments in this paper that depend on this separation should be read accordingly.

The most celebrated concrete example of quantum advantage that does not rely on this conjecture is (Shor's, 1994) algorithm for integer factorization, which achieves exponential speedup over the best-known classical algorithms and establishes that BQP contains pr}oblems believed to be outside BPP. Formal proofs of quantum advantage for specific problems have also been established in the shallow circuit model (Bravyi et al., 2018), providing unconditional (non-oracle) separations between classical and quantum computation that do not rely on unproven complexity assumptions.

The quantum supremacy experiment conducted by Google (Arute et al., 2019) in 2019, situated within the complexity-theoretic framework formalized by Aaronson and Chen (2017), demonstrated sampling from a 53-qubit quantum circuit distribution implementing a pseudo-random quantum circuit of the type analyzed by Boixo et al. (2018). Performance characteristics were as follows: one million samples were generated from the target distribution in 200 s. Classical verification of these samples using the best available tensor network methods (Pan and Zhang, 2022) was estimated to require approximately 10^4^ years of computation on contemporary supercomputers, although subsequent algorithmic improvements reduced this estimate to several days—illustrating how the classical-quantum frontier is actively contested. These results were extended to generative tasks by Huang et al. (2025) in a 2025 preprint reporting experiments on a 68-qubit processor; while not yet peer-reviewed, this work provides additional empirical support for the conjecture.

In addition to sampling, there are examples of quantum algorithms for linear algebra primitives, such as principal component analysis (Lloyd et al., 2014), support vector machines (Rebentrost et al., 2014), recommendation systems (Kerenidis and Prakash, 2017) and linear systems of equations (Harrow et al., 2009), which could be used in data-intensive applications in general. But the dequantization results from Tang (2019) showed that a number of these proposed exponential speedups can be matched with quantum inspired classical algorithms with sampling access to the data, significantly dampening the touted benefits. This type of work emphasizes the need to make careful claims of quantum advantage and to be able to differentiate oracle-relatives from worst-case separations.

Current technological restrictions do not contribute significantly to this discrepancy, however possible factor(s) include quantum state space characteristics. There is still some uncertainty as to whether this boundary constitutes a fundamental barrier or an engineering barrier since research in this area continues to evolve. This imbalance is not a short-term technological one. In contrast, a classical computer needs to explicitly list or statistically sample the probability distributions of classical memory structures. In quantum computers, probability distributions are instantiated in physical systems, and quantum superposition accesses the phenomenon of interference which creates them directly. The superposition principle and the exponential size of quantum state spaces form a representational advantage which no classical system can achieve without the exponential overhead.

### 3.3 Mathematical framework for quantum-classical separation in generative tasks

One of the major application fields of quantum computation was found as early as in the seminal work of Aspuru-Guzik et al. (2005) to be the calculation of the ground state properties of molecular Hamiltonians as detailed in Equation 2 (Cao et al., 2019). The minimum energy of a molecular system in which there are N electrons and M nuclei is given by:


$$\begin{array}{l} E_{0}\;=\;\min\text{\_}\left\{\psi \;\in \;H\right\}\;\langle \psi |\hat{H}|\psi \rangle \;/\;\langle \psi |\psi \rangle ,\\ \;\text{derived from} \end{array}$$ (2)

Cao et al., (2019)

where H represents the molecular Hamiltonian operator governing electron-electron and electron-nuclear interactions, and H denotes the fermionic Fock space of antisymmetric quantum states satisfying the Pauli exclusion principle.

In the case of an N-electron system coupled to K spatial orbitals, the dimension of the Hilbert space is proportional to C(2K, N), which increases exponentially with the size of the system, for any fixed ratio of electrons to orbitals. A Hilbert space containing a moderate organic molecule with 50 electrons has a dimension that is greater than the number of atoms in the observable universe (10^∧^80). This is not a limitation of the hardware for classical computers; studies such as the comprehensive reviews for quantum algorithms for chemistry and materials science (Bauer et al., 2020; McArdle et al., 2020) show that this is the fundamental barrier that drives toward quantum approaches.

This exponential difficulty can be overcome by using classical computational chemistry methods which use different approximation schemes, each of which has its own limitations in accuracy:

The advantages of Hartree–Fock theory are that it is a mean-field theory, and hence the scaling is *O*(*N*^4^), whereas the disadvantages are that it does not include dynamic electron correlation effects and the bond dissociation energy errors are 50–100 kcal/mol.

The full configuration interaction (Full-CI) methods systematically incorporate the effect of electron correlation by considering all the possible electronic configurations, but scale factorial *O(N!)* in the number of basis functions and are not realizable in practice unless the basis size is small. An alternative to complete CI is the use of classical neural networks to represent a quantum many-body wavefunction (Gao and Duan, 2017; Carleo and Troyer, 2017), which has the advantage of having much shorter representations but is still approximate and not efficient for strongly correlated systems.

Quantum algorithms can provide a way around these limitations. Quantum Phase Estimation (QPE) is provably polynomial (O(poly(N, ε^−1^))), assuming an efficiently preparable trial state that has non-negligible overlap with the ground state (Cao et al., 2019). There are heuristic alternatives, such as the Variational Quantum Eigensolver (VQE) (Peruzzo et al., 2014; Kandala et al., 2017) and hardware-efficient variants, which rely on the quality of the ansatz and the optimization landscape and have performance that is not guaranteed to scale with a polynomial factor; in contrast to QPE, VQE has no proven polynomial scaling and suffers from the barren plateaus problem (discussed in Section 6). It is crucial to make this distinction between the scaling of QPE and the heuristic approach of VQE, and it should not be confused. The polynomial scaling of QPE, shown in Equation 3,


$$\begin{array}{l} O\left(poly\left(N,\;\varepsilon^{-}\right)\right) \end{array}$$ (3)

where ε denotes the target accuracy, represents a genuine advantage for strongly correlated quantum systems where classical approximations fail.

The complete specification of an N-electron molecular wavefunction in the configuration interaction framework requires exponentially many complex coefficients. The wavefunction expands on the basis of Slater determinants as:


$$\begin{array}{l} |\psi \rangle =\sum_{\text{I=1}}^{\text{C}(\text{2K},\text{N})}c_{\text{I}}|D_{\text{I}}\rangle ,{\sum_{\text{I=1}}^{\text{C}(\text{2K},\text{N})}\left|c_{\text{I}}\right|}^{2}=1,\\ \text{ derived from} \end{array}$$ (4)

Cao et al., (2019)

Here, each Slater determinant |D_I〉 describes a different electronic configuration, and the normalization condition defined in Equation 4 guarantees that that the probabilities are unity. A classical representation of this kind for a system of *N* = 100 electrons in *K* = 200 spatial orbitals would involve the storage of the approximate value of 10^∧^60 complex coefficients. This representation would still need more information capacity than could be physically encoded in all matter of the observable universe if each coefficient could only be stored in one (bit) piece of information. This exponentially large information content is stored in only 2K = 400 qubits in a quantum computer, via physical superposition; it is native, and can be compacted without approximation or information loss.

### 3.4 Effects of generative AI architectures

The restrictions are not that classical generative AI is not suitable in general, but rather that are where it is not suitable. This analysis shows that there are three simple rules that govern the evolution of quantum enhanced generative systems.

- First, as stated above, the natural domains of data are located well within the classical computational realm. Large language models are currently the predominant technology in text generation, image synthesis, video production, and speech synthesis, and are expected to remain so for tasks grounded in natural-data distributions. Architectures like BERT (Devlin et al., 2019) and GPT-3 (Brown et al., 2020) have shown that transformer-based models are capable of state-of-the-art performance in almost every natural language task trained on natural data. These inductive biases—locality by the attention window, composition by a stack of layers, and learned positional encodings—are well known in the foundation model's literature (Bommasani et al., 2021) and are well suited to the statistical regularities in natural language, visual scenes and acoustic signals. The problem is that there is no quantum advantage in these problems since the underlying probability distributions are not a result of a quantum mechanical process, but are a result of a classical stochastic process, which represents the mechanism of human communication and perception.
- Second, for efficient processing of quantum-native computational tasks, quantum backends are needed. Probability distributions arise naturally in the realm of quantum physics in molecular design, quantum simulation, material discovery, quantum circuit synthesis and optimization of quantum error correction codes. Classical generative models can learn approximations of these distributions from a finite training set, but cannot efficiently sample from the actual distributions or make exact inference without exponential extra cost. These tasks have a quantum mechanical nature, and require quantum computational resources not for their computational convenience but for their quantum nature.
- Thirdly, hybrid classical-quantum architectures are identified as the best option. Classical models supply high level reasoning abilities, natural language interfaces, and knowledge representation, while quantum systems are used as specialized coprocessors, only when classical systems hit fundamental efficiency limits on sub-tasks. The principal component of this coprocessor role is parameterized quantum circuits (PQCs) (Benedetti et al., 2019) which are trainable quantum circuits that may be optimized together with classical networks. The capacity of PQCs (Du et al., 2020) to represent complex probability distributions is a major theoretical drive for their use as quantum generative models.

Table 1 summarizes the capabilities of the various architectures, highlighting the strengths of each approach at a specific task.

**Table 1:** Comparison of classical, NISQ and hybrid architectures.

Property	Classical (LLM/GAN)	Quantum (NISQ)	Hybrid
Natural language tasks	Excellent	Not suitable	Classical handles NL
Quantum sampling	Exponential cost	Polynomial *O(d)*	Quantum subsystem
Molecular simulation	Exponential (Full-CI/*O(N!)*)	Polynomial (QPE, proven)	Quantum coprocessor
Unstructured search	*O(N)*	*O*(√*N*) via Grover	Quantum search module
Hardware maturity	Production-ready	NISQ-limited	Classical dominant
Error tolerance	High	Low (0.1%−1% gate error)	Errors isolated to quantum subroutine
Trainability	Gradient descent (proven)	Barren plateaus (heuristic)	

## 4 Hybrid approach to successes

**Figure 2:**
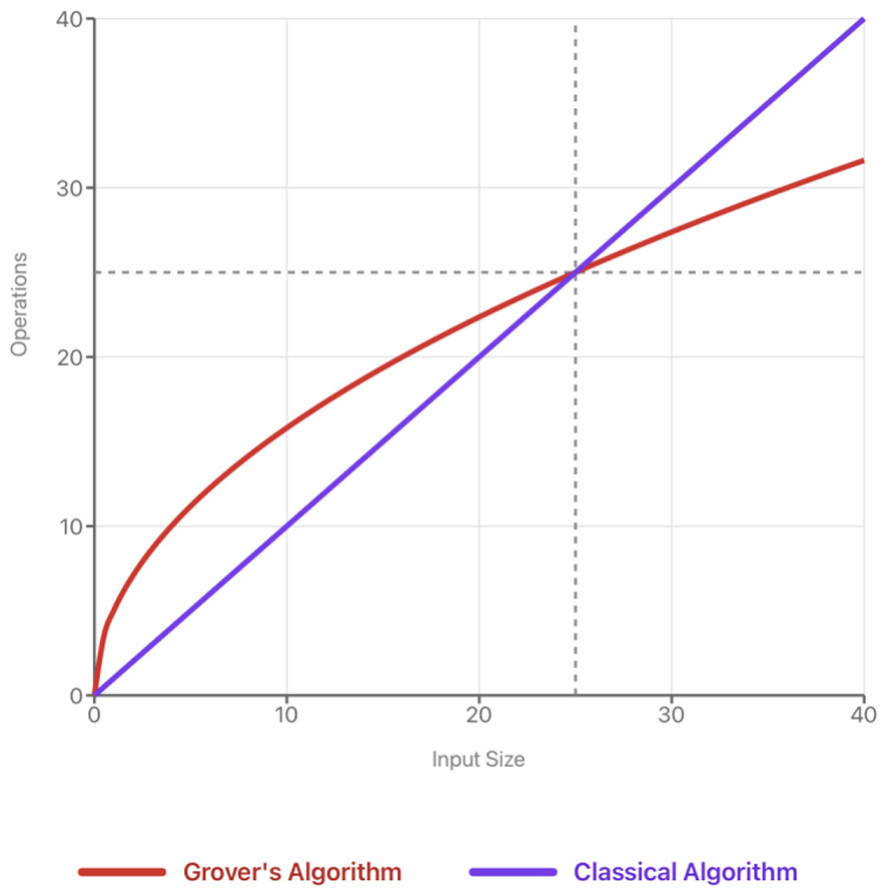
Comparison of computational operations required by classical search methods vs. the Grover Quantum Search (GQS) algorithm as a function of input size N.

Quantum computing integrates into generative AI systems not as a replacement but as a coprocessor handling specific tasks where classical methods face fundamental efficiency barriers. Variational quantum algorithms (Cerezo et al., 2021) represent the primary paradigm for near-term quantum computation, encompassing VQE, QAOA, and related approaches. Quantum-enhanced feature spaces (Havlíček et al., 2019) represent a complementary approach in which the kernel of a support vector machine is computed using a quantum circuit, potentially providing advantage for classically intractable data distributions. In Figure 2 shows a classical method and an algorithm—GQS Algorithm comparison for the operations and input sizes. Case studies have been created for a tangible example where quantum systems provide strong theoretical benefits from using a quantum-enhanced hybrid pipeline.

### 4.1 Case study: optimizing rag using Grover's search algorithm

Retrieval-Augmented Generation (RAG) (Lewis et al., 2020) is an extension that seeks to enhance the capabilities of LLMs by dynamically accessing external documents of a knowledge base to answer queries it has not seen during training. This model is the basis for numerous practical applications of LLM. The typical RAG pipeline consists of three parts: query encoding, document retrieval, and conditioned generation. The second step—document retrieval—is a computational challenge for classical methods are appropriate at the case of billions of candidates. Suppose we have a knowledge base D = {d1, d_2_, ..., d_N_} with N documents and each document d_i_ has an embedding vector e_i_ ∈ℝ^∧^k in the learned semantic space. The retrieval task is to find the subset S of documents, S ⊆ D, that meet a relevance criterion, usually a similarity threshold:


$$\begin{array}{l} S\;=\;\left\{di\;:\;sim\left(q,\;ei\right)\;>\;\tau \right\}\left(5\right),\\ \text{derived from} \end{array}$$

Lewis et al., (2020)

where sim denotes a similarity metric (cosine similarity or dot product) and τ represents the relevance threshold.

Classical implementations of this retrieval operation use approximate nearest-neighbor (ANN) search algorithms. Modern ANN methods—such as hierarchical navigable small world (HNSW) graphs (Malkov and Yashunin, 2020) and learned index structures (Kraska et al., 2018)—already achieve O(log N) or sub-logarithmic query time on structured, preprocessed data. It is therefore critical to scope the quantum advantage claim carefully: Grover's algorithm provides quadratic speedup over linear classical search for genuinely unstructured or highly dynamic databases where index construction is infeasible or prohibitively expensive. For well-structured static databases, modern ANN methods may already outperform the O(√N) Grover bound. The hybrid RAG proposal in this paper targets precisely the unstructured or adversarially dynamic regime. For databases containing N = 10^9^ documents operated under dynamic update conditions—such as enterprise search applications or LLM-integrated question-answering systems—where classical index maintenance costs become computationally prohibitive, the classical worst-case search complexity reverts to Θ(N) evaluations, and the latency impact on user experience is substantial.

Grover's (1996) quantum search algorithm provides a quadratic speedup for such unstructured search problems by locating marked database entries in O(√N) queries. The algorithm constructs a quantum circuit that iteratively amplifies the amplitude associated with marked database entries through repeated application of the Grover operator. The mathematical structure of amplitude amplification can be geometrically understood as rotations in a 2D subspace spanned by the uniform superposition over unmarked states and the marked states. After approximately ⌊(π/4)√(N/M)⌋ iterations, where M denotes the number of marked items satisfying the search criterion, measurement of the quantum register yields a marked index with probability exceeding 1 - O(M/N).

The Grover operator G decomposes into two reflections: phase inversion about marked states implemented through the oracle O_f_, and inversion about the average amplitude defined in Equation 6:


$$\begin{array}{l} \;G\;=\;(2|\psi \rangle \langle \psi |\;-\;I),\\ \text{derived from} \end{array}$$

**Figure 3:**
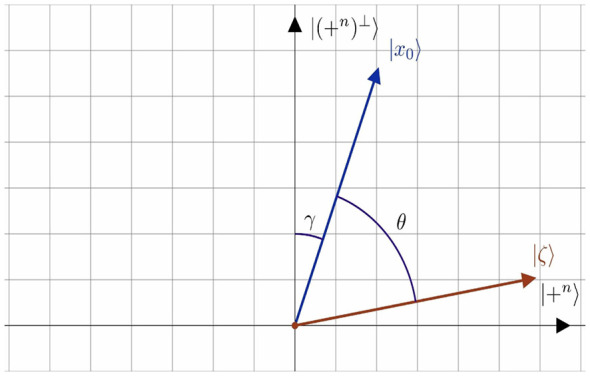
Geometric interpretation of Grover's amplitude amplification, showing the state vector rotating toward the marked state |α〉 with each iteration of the Grover operator.

Grover's (1996)

where |ψ 〉 = (1/√N) Σ_{x=0}^∧^{N-1} |x〉 represents the uniform superposition state prepared through Hadamard gates applied to all qubits initially in state |0〉, and the oracle O_f|x_〉 = (−1)^∧^{*f(x)*}|x〉 with *f(x)* = 1 for marked entries and *f(x)* = 0 otherwise. Figure 3 shows that the amplitude amplification process can be geometrically understood as a rotation of the state vector closer to the marked state |α〉 with each iteration.

**Figure 4:**
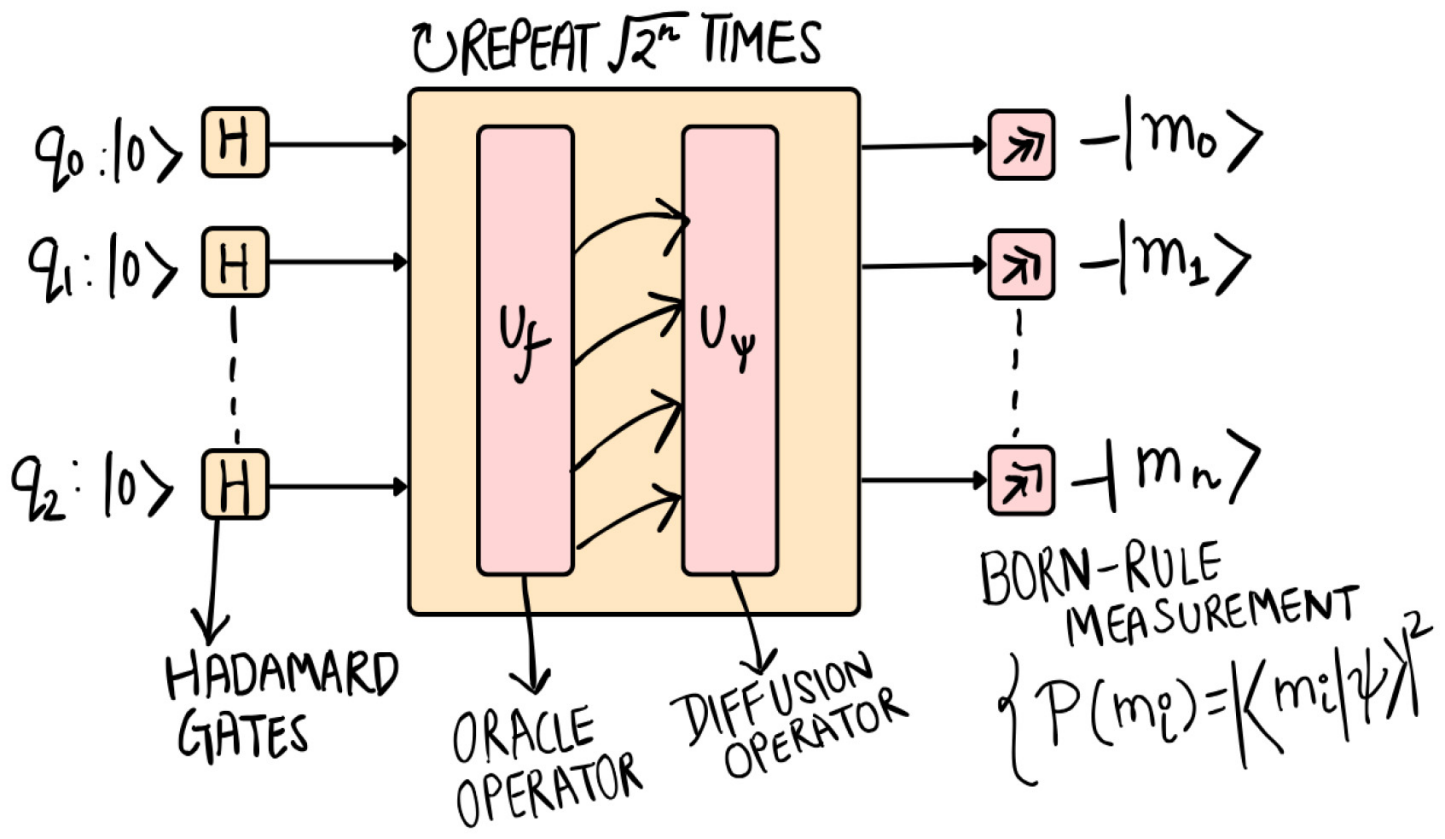
Quantum circuit implementation of Grover's Search Algorithm (GSA), showing the Hadamard layer, phase oracle, and diffusion operator repeated O(√N) times.

The diffusion operator performs inversion about the average amplitude, constructively interfering to amplify marked state amplitudes while suppressing unmarked state amplitudes through destructive interference. Figure 4 shows the practical quantum circuit implementation, illustrating the arrangement of Hadamard gates H^∧^{⊗n}, the phase oracle U_f_ (marking target states), and the diffusion operator U_ψ_ (inverting about the mean amplitude), repeated *O*(√*N*) times.

The complete hybrid architecture works as follows when integrated to RAG pipelines: The classical language model encoder takes as input the user's natural language query and outputs the semantic embedding q ε R^k.^

- This embedding vector is sent to the quantum subsystem and stored in the amplitude of the quantum states through amplitude encoding. The formal definition of amplitude encoding needs a poly(log *N, k)* quantum gate, but this is assuming the availability of an efficient quantum random access memory (QRAM), which is currently not available in practice at large scale (Jaques and Rattew, 2023). This is an important open problem for any envisaged system that is intended to be implemented in near future.
- The quantum processor performs Grover's algorithm using a similarity oracle that represents the relevance criterion in Equation 5 which requires *O*(√*N)* iterations with each iteration calling the oracle circuit.
- The quantum register operates over a superposition of all possible states, enabling a quadratic speedup in identifying the desired result relative to classical linear search—subject to the practical constraints on QRAM and gate fidelity discussed in Section 6.
- Measurement results (document indices) are fed back into the classical system which downloads the document content from the storage and brings it to the generation stage.
- With this classical LLM, a recovered context and the original user query, the LLM creates the desired natural language output.

In terms of practical considerations for implementation, there is quantum state preparation overhead (O(k log N) operations needed to embed dimension k in a quantum computer; this is a one-off operation for a static database), quantum measurement errors and gate noise (requiring quantum error mitigation strategies (O'Brien et al., 2023) in the NISQ era), and classical quantum communication latency (as described in Section 6).

Although the algorithm has its drawbacks, Grover's algorithm is theoretically very promising for unstructured search. It would require significant leaps forward in the technology of quantum error correcting codes, coherence times, and practical implementations of QRAM architectures before this method could be used to create a production ready RAG system.

## 5 Current limitations and future outlook

While the potential for quantum-enhanced generative pipelines is promising, there are several challenges that still need to be addressed. These challenges involve algorithm design, fabrication of hardware and integration into a system.

### 5.1 Algorithmic challenges: barren plateaus

The barren plateau phenomenon (McClean et al., 2018; Larocca et al., 2025) is a fundamental algorithmic challenge for many quantum algorithms, including for variational algorithms such as the Variational Quantum Eigensolver (VQE) (Farhi et al., 2014) and the Quantum Approximate Optimization Algorithm (QAOA) (Farhi et al., 2014) and quantum neural network classifiers (Farhi and Neven, 2018). The spread of the gradients of the cost functions associated to a randomly initialized parameterized quantum circuit is exponentially smaller with circuit depth and number of qubits. The variance of the gradient is proportional to n for n qubits. For circuits with n qubits, the gradient variance scales as:


$$\begin{array}{l} Var\left[\partial_{\text{θ}}E\right]\sim O\left(2^{-\text{n}}\right)\left(7\right),\\ \text{derived from} \end{array}$$

(McClean et al., 2018)

The issue is exacerbated by hardware noise: noise induced barren plateaus (Wang et al., 2021) can lead to gradient vanishing, even in shallow circuits, irrespective of random initialization. Examples of mitigation techniques involve LLM inspired initialization schemes of parameters that have been demonstrated to enhance gradient variance in quantum neural networks (Zhuang and Guan, 2025), structured ansatz design and layer-wise training approaches (Larocca et al., 2025). But, barren plateaus limit the trainability of quantum circuits in a mathematically interesting way, which continues to be a research topic.

### 5.2 Hardware challenges: NISQ limitations

Today's noisy intermediate-scale quantum (NISQ) processors (Preskill, 2018) have significant restrictions on their practical use. Superconducting-qubit-based systems also yield an error rate of 0.1%−1%, and a coherence time of 100 μs to several milliseconds, for 50–1,000 physical qubits, which is far beyond the fault-tolerance requirement for quantum error correction (QEC) (Gottesman, 2010). An alternative hardware approach is the use of photonic platforms, with recent experimental demonstrations of programmable nanophotonic devices that can perform multiple photon quantum circuits (Arrazola et al., 2021) but coherence and scaling are yet to be achieved.

Error-free quantum computation using a practically useful number of logical qubits (*n* = 10^3^−104) will only be possible if the gate error rates are below about 0.01% (Gottesman, 2010). Meanwhile, near-term error mitigation techniques (O'Brien et al., 2023) (e.g., probabilistic error cancellation and purification-based methods) provide limited near-term respite for some quantum chemistry calculations, but with polynomial overhead in the number of circuit evaluations. Recent experiments with utility scale quantum processors have yielded evidence that quantum circuits can solve problems currently unsolvable in an exact simulation in classical computers, even prior to achieving full fault-tolerance (Kim et al., 2023), which means that near-term hybrid systems will have some practical use sooner than the fault-tolerance timelines suggest.

With the advances being made in this area, quantum systems are expected to outperform classical supercomputers for certain applications best suited to quantum computing within the next 10–15 years, subject to a number of investment, materials science and error correction advances.

### 5.3 System integration challenges

Efficient integration of quantum coprocessors with classical AI infrastructure introduces latency as a primary obstacle. The latency of classical-quantum round trips for variational algorithms currently dominates execution time, and cloud-based quantum computing services offered by IBM, Google, and Amazon introduce additional communication delays that are incompatible with real-time inference or training applications. Another challenge that stands out is the lack of efficient quantum random access memory (QRAM) architectures (Jaques and Rattew, 2023) for application such as RAG system described in Section 4. Compounding this, Menickelly et al. (2023) show that the optimal stochastic optimizer for variational quantum algorithms depends on the ratio of circuit switching latency to the time required to execute shots, such that the optimizers are not just a deployment consideration, but also important for algorithmic efficiency during training.

**Figure 5:**
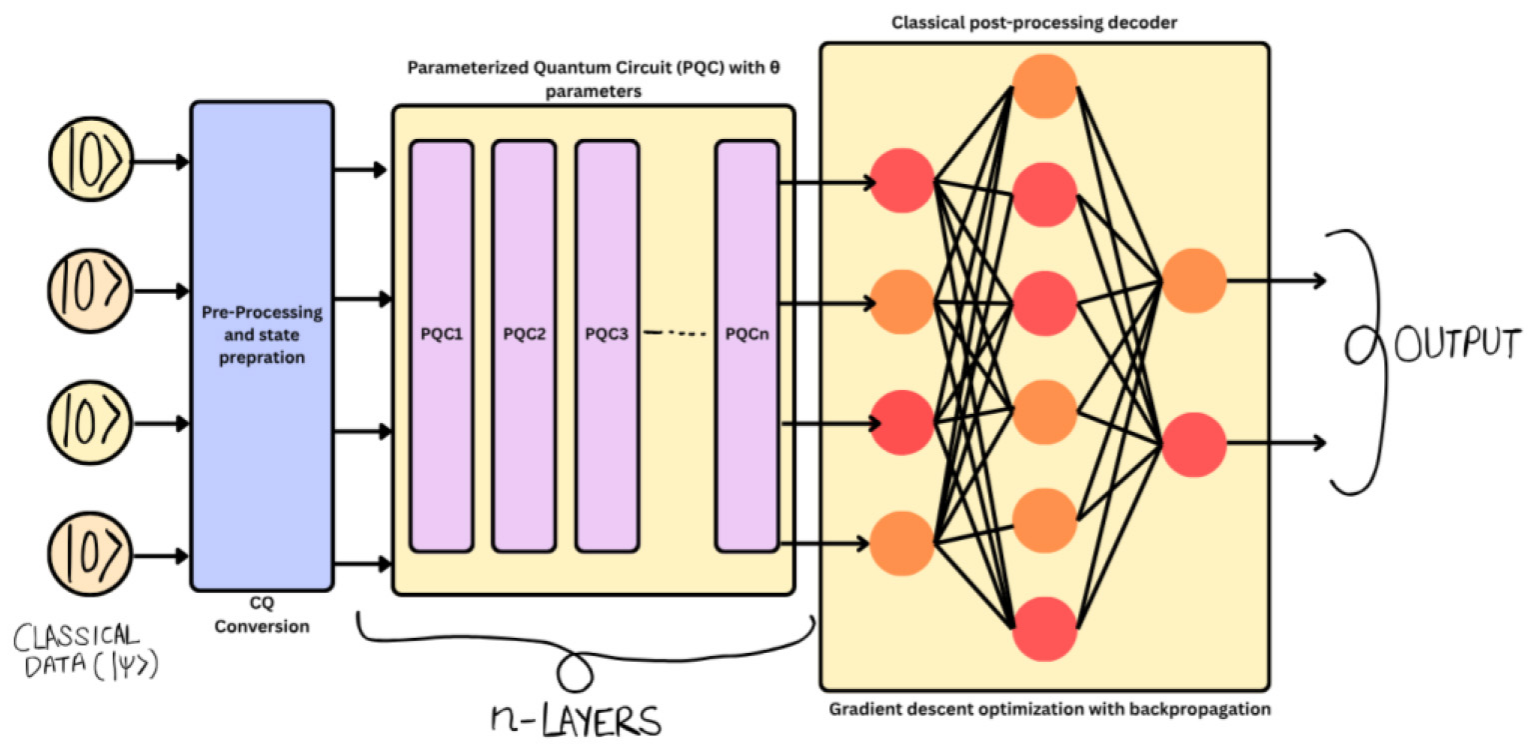
Proposed hybrid QCN-CNN architecture where a parameterized quantum circuit (PQC) acts as a feature-mapping pre-processor, passing measurement outputs to a classical CNN decoder for inference.

Figure 5 proposes a hybrid quantum-classical neural network (QCN) architecture to mitigate these integration challenges, where a parameterized quantum circuit (PQC) acts as a quantum feature-mapping pre-processor, encoding classical input data into a high-dimensional Hilbert space. The quantum measurement output is passed to a classical convolutional neural network (CNN) decoder for downstream inference tasks.

Current Variational Quantum Algorithms possess a latency problem because of Classical-Quantum Communications. Furthermore, all present-day cloud-based Quantum Computing service providers such as IBM, Google and Amazon further contribute to this latency and thus, are not currently able to offer real-time inference and training applications. Recent experiments demonstrate that the ratio of latency to number of shots taken by an optimizer is an important performance metric from which to assess optimizer performance as well as to implement effective variational quantum algorithms (Menickelly et al., 2023).

## 6 Discussion

A number of pressures have emerged from this literature that are worthy of explicit discussion in this review.

On the hybrid RAG proposal: Section 6 presents a conceptual contribution of the hybrid RAG system, which is based on the existing results for Grover's (1996) algorithm and amplitude encoding. To do this, one needs to have a working QRAM architecture (Jaques and Rattew, 2023) and oracle circuits that are reversible for cosine similarity, as well as gate error rates much below the NISQ error thresholds (Preskill, 2018). At production scale, none of these are available at this time. The proposed plan is therefore not a system that needs to be deployed immediately but rather a roadmap for the near future.

In terms of the quantum advantage of RAG. Modern ANN methods (Malkov and Yashunin, 2020; Kraska et al., 2018) already have sub-linear query time for structured data as mentioned in Section 6. The quantum speed-up only has relevance for unstructured or adversarially dynamic databases. The crossover point, i.e., the database size N where Grover's O(√N) advantage over linear classical search is greater than the quantum overhead [state preparation (Jaques and Rattew, 2023), measurement, and classical-quantum communication latency], should be quantified for future work.

On BPP ⊆ BQP. What has not been proved, however, is the separation that is the basis for much of the theoretical discussion of Section 3. While this conjecture is supported by empirical demonstrations of quantum supremacy (Arute et al., 2019) it remains unclear. This separation is a disclaimer for all claims in this paper that rely on this separation, and authors of future works making such claims are encouraged to be explicit as to whether they are unconditional (such as Bravyi et al., 2018; Shor, 1994) or whether they depend on a conjecture.

On dequantization. The success of Tang (2019) and related work clearly shows that the performance of some proposed quantum speedups can be achieved via the quantum-inspired classical algorithm in the right data access scenario. It is important to take into account whether the resulting speedup remains in practice when considering reality in the data access model of any future proposal for a quantum advantage in a data processing task.

These restrictions do not detract from the merit of the hybrid quantum-classical framework introduced here, but rather delineate some engineering and theoretical goals that need to be achieved before this framework can be realized.

## 7 Conclusion

In this review, three questions have been discussed, in order. Firstly, why quantum neural networks (QNNs) cannot directly replace existing classical networks used in tasks such as natural language processing or image synthesis, and what they can achieve. The areas of quantum-based solutions that will be useful are those areas where classical computation has fundamental mathematical limitations, where a quantum mechanical probability distribution would be needed, where calculation of the electronic structure of a molecule would be needed or where an exponentially large combinatorial optimization space would be required.

Secondly, a look was taken at how these solutions would be implemented. The formal results and empirical demonstrations of how probability distributions exhibit computational difficulty for classical algorithms because of the mathematical framework underlying information theory (rather than hardware failures), along with a conjectured separation of BPP from BQP, provide evidence that the use of classical computational methods encounter barriers based on those conditions. This does not prove the conjectured separation of BPP from BQP but does support it by showing that when attempting to use classical algorithms to solve problems based on certain defined probability distributions, very significant differences exist. Hence, quantum computing has been shown to not be competing with classical artificial intelligence but is a coprocessor that can assist in solving problems that fall into different categories. Through this, quantum computing is established not as a competitor to classical AI but as a coprocessor addressing distinct problem classes.

Third, the reasons why quantum systems are not yet widely adopted were examined. Barren plateau phenomena (McClean et al., 2018; Larocca et al., 2025) limit the trainability of variational quantum circuits. NISQ-era quantum processors (Preskill, 2018) provide 50–1,000 noisy qubits with gate error rates of 0.1%−1%, which is too high for fault-tolerant computation. In RAG applications, data encoding overhead—compounded by the absence of efficient QRAM architectures (Jaques and Rattew, 2023)—frequently negates theoretical quantum speedups for modest dataset sizes.

The hybrid classical-quantum architecture is the optimal near-term architecture for integrating quantum resources into generative AI systems. The most effective implementations use classical models for high-level reasoning and natural language processing while invoking quantum coprocessors only for specific subtasks—molecular simulation (Aspuru-Guzik et al., 2005; Bauer et al., 2020; McArdle et al., 2020), unstructured search (Grover, 1996), and quantum-native generative modeling (Coyle et al., 2020)—where quantum resources provide a well-founded advantage. Other important future application domains include quantum-accelerated financial modeling (Egger et al., 2020; Pistoia et al., 2021) and large-scale drug discovery (Cao et al., 2018).

The convergence of quantum and classical architectures through hybrid systems is the most scientifically grounded and practically viable path toward expanding generative AI capabilities into domains that are currently inaccessible to purely classical approaches. Quantum natural language processing (Nausheen et al., 2025) remains an open research frontier, and the maturation of quantum hardware will progressively expand the scope of tasks for which hybrid integration is worthwhile.
